# Short term supplementation of dietary antioxidants selectively regulates the inflammatory responses during early cutaneous wound healing in diabetic mice

**DOI:** 10.1186/1743-7075-8-80

**Published:** 2011-11-17

**Authors:** Na-Young Park, Yunsook Lim

**Affiliations:** 1 Department of Food and Nutrition, Kyung Hee University, Seoul 130-701, Republic of Korea

**Keywords:** Diabetes, antioxidant, inflammation, oxidative stress, wound healing

## Abstract

**Background:**

Diabetic foot ulcers are serious complications for diabetic patients, yet the precise mechanism that underlines the treatment of these diabetic complications remains unclear. We hypothesized that dietary antioxidant supplementation with vitamin C, combined either with vitamin E or with vitamin E and NAC, improves delayed wound healing through modulation of blood glucose levels, oxidative stress, and inflammatory response.

**Methods:**

Diabetes was induced by administration of alloxan monohydrate. Mice were divided into 4 groups; CON (non-diabetic control mice fed AIN 93 G purified rodent diet), DM (diabetic mice fed AIN 93 G purified rodent diet), VCE (diabetic mice fed 0.5% vitamin C and 0.5% vitamin E supplemented diet), and Comb (diabetic mice fed 0.5% vitamin C, 0.5% vitamin E, and 2.5% NAC supplemented diet). After 10 days of dietary antioxidant supplementation, cutaneous full-thickness excisional wounds were performed, and the rate of wound closure was examined. TBARS as lipid peroxidation products and vitamin E levels were measured in the liver. Expression levels of oxidative stress and inflammatory response related proteins were measured in the cutaneous wound site.

**Results:**

Dietary antioxidant supplementation improved blood glucose levels and wound closure rate and increased liver vitamin E, but not liver TBARS levels in the diabetic mice as compared to those of the CON. In addition, dietary antioxidant supplementation modulated the expression levels of pIκBα, HO-1, CuZnSOD, iNOS and COX-2 proteins in the diabetic mice.

**Conclusions:**

These findings demonstrated that delayed wound healing is associated with an inflammatory response induced by hyperglycaemia, and suggests that dietary antioxidant supplementation may have beneficial effects on wound healing through selective modulation of blood glucose levels, oxidative stress, and inflammatory response.

## Introduction

Diabetes mellitus (DM) is a disease in which injury of peripheral tissue is induced by oxidative stress caused by chronic hyperglycaemia. The number of DM patients is now approximately 250 million people and is expected to reach 400 million by 2025 [1]. It is known that the development of diabetes and its complications are accompanied by an increase in oxidative stress and inflammatory response. In particular, foot ulcers are one of the major complications of diabetes, caused by neuropathic and vascular complications. Mortality from diabetes is mainly associated with foot ulcers and amputations, which remain common, along with serious complications, including an impaired wound healing process.

During the inflammatory stage, the first stage in the wound healing process, neutrophils and macrophages infiltrate the wound site and phagocytose infectious agents and fragments of tissue degradation release proteases and various reactive oxygen species (ROS) into the wound environment [2]. They also are both major sources and targets of pro-inflammatory cytokines such as IL-1β and TNF-α, which have been shown to be key mediators by promoting NFκB activation and ROS production during cutaneous inflammatory processes [3]. ROS plays crucial roles in cell signaling and immune response but causes oxidative stress at higher levels during wound healing. Therefore, regulation of oxidative stress and inflammatory response is an important factor in cutaneous wound healing.

Diabetic patients have impaired wound healing, which is considered as chronic and delayed healing if it continues beyond 8 weeks [4]. They are thus more prone to develop foot ulcers resulting from a variety of factors including a peripheral circulation failure, modified leukocyte function and cytokine production, and even chronic hyperglycaemia itself [5-7]. Delayed wound healing in diabetes not only reduces insulin sensitivity and the occurrence of glycosylation of various proteins, enzymes, and insulin caused by hyperglycaemia, but also raises oxidative stress and decreases antioxidant defense systems [8,9]. Furthermore, ROS production caused by wounds themselves aggravates oxidative stress accompanied by a deteriorated antioxidant condition. To improve wound healing and healing of foot ulcers in diabetes, a proper understanding of mechanisms leading to diabetic ulcers is required. At the cellular levels, previous studies observed an absence of the transforming growth factor (TGF)-β1 and the insulin-like growth factor (IGF)-1, as well as increased levels of matrix metalloproteinases and decreased levels of their inhibitors [7]. The levels of pro-inflammatory cytokines and inflammatory mediators, such as TNF-α, IL-6, iNOS, and COX-2, were increased in diabetes [10-13]. The abnormal metabolism of diabetes causes insufficient migration of inflammatory cells to the wounds, accompanied with decreased chemotaxis of leukocytes [14], which is contributory to delayed wound healing in chronic diabetic foot ulcers. Therefore, there are highly infectious risks caused by retarded or chronic inflammatory responses of wounds in diabetic patients. In previous studies, diabetic wounds showed lower levels of catalase, glutathione (GSH), vitamin C, and vitamin E [15]. In addition, supplementation with N-acetylcysteine (NAC), a precursor of GSH, attenuated the severity of diabetes and inhibited the over-activation of NFκB [16], and GSH treatment aided the delayed healing process in diabetes [15]. α-tocopherol reduced plasma malondialdehyde (MDA) levels, increased activities of antioxidant enzymes, such as superoxide dismutase (SOD) and GSH peroxidase, and promoted the wound healing process [17,18]. Based on the previous studies, the beneficial effects of a single antioxidant nutrient were limited in diabetes or wound occurrences. Also, little research using a dietary antioxidant combination as a modulator of early inflammatory response during the wound healing process has been conducted. We hypothesize that a combination of antioxidant nutrients, which have previously shown synergetic effects, can accelerate delayed cutaneous wound healing through a modulation of inflammatory responses.

## Methods

### Diabetes induced Animals

Female ICR mice (5.5 weeks old) were purchased from the Central Lab. (Seoul, Republic of Korea). The mice were individually housed in polycarbonate shoebox type cages with wire tops in a room maintained at 22 ± 1°C and 50 ± 1% humidity on a 12-h light/dark cycle, with free access to water and chow diet for 1 week. Diabetes was induced by alloxan monohydrate (180 mg/kg body weight, i.p. injection; Sigma-Aldrich, St Louis, MO, USA) in 0.9% NaCl. Non-diabetic control mice were injected with saline. After 5 days, the induction of diabetes was confirmed by measuring fasting blood glucose levels and monitoring for 10 days. Mice with a fasting blood glucose level ≥ 250 mg/dl were used for this study. Glucose levels were measured by a one-touch blood glucose meter (LifeScan Inc., Milpitas, USA) from the tail vein at the same time to minimize the effect of diurnal fluctuation. All mice were used in accordance with animal protocols approved by the Kyung Hee University Institutional Animal Care and Use Committee.

### Experimental diets

Mice were divided into 4 groups that were fed different antioxidant supplementation; i) Group 1 (CON): non-diabetic control mice were fed AIN 93 G Rodent purified diet; ii) Group 2 (DM): diabetic mice were fed AIN 93 G Rodent purified diet; iii) Group 3 (VCE): diabetic mice were fed 0.5% vitamin C (L-ascorbic acid, > = 99.0%, Crystalline; Sigma-Aldrich) and 0.5% vitamin E ((+/-) α-tocopherol, synthetic, > = 96%; Sigma-Aldrich); and iv) Group 4 (Comb): diabetic mice were fed 0.5% vitamin C, 0.5% vitamin E, and 2.5% NAC (N-acetyl-L-cysteine, Sigma grade, > = 99%, Sigma-Aldrich) for 10 days.

### Sample collection and preparation

Blood, wounds, and livers at 0 h (basal level), 24 h, 48 h, and 72 h, after wounding, were collected. Blood samples were taken using a 1 cc syringe with heparin and centrifuged at 3000 rpm for 10 min at 4°C. All samples were kept at -80°C until processed.

### Measurement of lipid peroxidation product in liver

The liver thiobarbituric acid reacting substances (TBARS) level was measured as an index of lipid peroxidation [19]. Briefly, liver homogenates were prepared in a 0.15 M potassium chloride buffer. A sample (200 ㎕) was added to 200 ㎕ of 8.1% SDS and placed in room temperature for 10 minutes, and 3 ㎖ of a 20% acetic acid-0.8% thiobarbituric acid (TBA) mixture and 600 ㎕ of distilled water were added. The mixture was heated at 95°C for 1 hour in a boiling water bath. After cooling, a mixture of 1 ㎖ distilled water and 5 ㎖ of n-butanol and pyridine was added and shaken vigorously. After centrifuging at 4000 rpm for 10 minutes, the light absorbance of the supernatant was measured at 532 nm. 1,1,3,3-tetramethoxypropane was used as a standard and prepared at concentrations in the range of 0~180 nM (0, 40, 60, 120, 160, and 180 nM).

### Measurement of vitamin E level in the liver

The level of vitamin E in the liver was measured using a high-performance liquid chromatography (HPLC) system as previously described [20]. The mouse liver was homogenized in a lysis buffer. 0.5% butylated hydroxytoluene (BHT) dissolved in methanol and hexan was added to the homogenized solution and centrifuged at 3200 rpm for 20 minutes at 4°C. Subsequently, the suspension was evaporated under a stream of nitrogen after which methanol was added. α-tocopherol was used as standard solution.

### Wound biopsy

Full thickness excisional wounds were made on the back of the mouse. The mice were, briefly, anesthetized with isoflurane (Aerane liquid, Ilsung pharmaceuticals Co., LTD, Seoul, Republic of Korea) and the backs of all mice were shaved using a hair clipper and then sterilized by 70% (v/v) ethanol. The wound biopsy model used in this experiment has been previously described [21]. The full-thickness excisional wounds were made on the folded skin by a sterile biopsy punch (4 mm diameter, Kai medical, Gifu City, Japan). The yielded two circular wounds on the dorsum, below the shoulder blades of each mouse were made to avoid self-licking.

### Measurement of wound closure rate

Wounds in each individual mouse were photographed digitally every day, beginning on the day of wounding (d 0) with a standard dot equivalent to the initial wound area placed beside the wound. The quantification of wound closure used in this experiment was previously described [22]. Wound closure was quantified by Canvas 11SE software (Deneba, Miami, FL, USA). The rate of wound closure was expressed as the ratio of wound area (each day after wounding) compared to the initial wound area. A smaller wound ratio indicated faster wound closure.

### Western blotting assay

Skin was homogenized in a lysis buffer (containing Trizma base, NaCl, 10% NP40, 10% Na-dedoxycholate, 100 mM EDTA, and 10% SDS) with a protease inhibitor (1:200 v/v, Sigma Aldrich) and centrifuged at 16,000 rpm for 30 minutes at 4°C. The sample (60 μg protein) was separated on 10% SDS PAGE gels and then transferred to the PVDF membrane. After blocking for 1 hour in 5% skim milk, the membranes were incubated with specific monoclonal and polyclonal antibodies against pIκBα (Santa Cruz Biotechnology, Santa Cruz CA, USA, 1:200), HO-1 (Stressgen, Victoria BC, Canada, 1:2000), CuZnSOD (Santa Cruz Biotechnology, 1:500), iNOS (Stressgen, 1:2000), COX-2 (Transduction Laboratories, Lexington KY, USA, 1:250) and β-actin (Santa Cruz Biotechnology, 1:800) overnight at 4°C, and then incubated with a secondary antibody for 1 hour at room temperature. The blots were detected by enhanced chemiluminescence and measured by the Image J program. β-actin was used for normalization of the target protein expression in each sample.

### Statistical analysis

All values are expressed as means ± SEM. Data were analyzed by 1-way ANOVA using SPSS (version 12) statistical analysis program, and then differences among means were analyzed using Duncan's test. The relationships between relative wound size and blood glucose levels were evaluated by Pearson's correlation coefficients. For all tests, differences were considered significant at *P *< .05.

## Results

### Body weights

The body weights of all diabetic mice were similar to those of the CON group regardless of antioxidant supplementation (data not shown).

### Blood glucose levels

**Figure 1:**
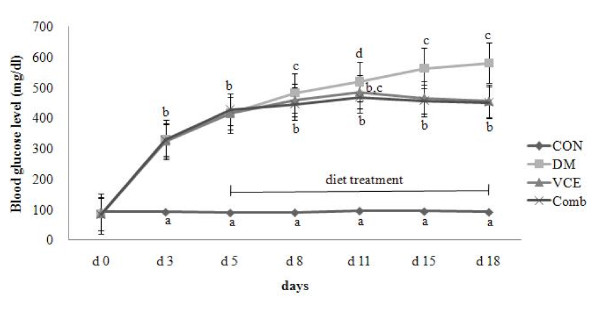
**Effect of dietary antioxidant supplementation on blood glucose level in alloxan-induced diabetic mice**. Values are means ± SEM. Means at a time without a common letter differ, *P *< .05. (CON, control mice; DM, diabetic control mice; VCE, 0.5% vitamin C and 0.5% vitamin E supplemented diabetic mice; Comb, 0.5% vitamin C, 0.5% vitamin E, and 2.5% NAC supplemented diabetic mice).

Blood glucose levels of all diabetic mice were significantly increased after alloxan injection. Although blood glucose levels of the VCE and the Comb groups did not go back to the steady levels, there was a significant reduction as compared to those of the DM group (Figure 1).

### Liver TBARS levels

**Figure 2:**
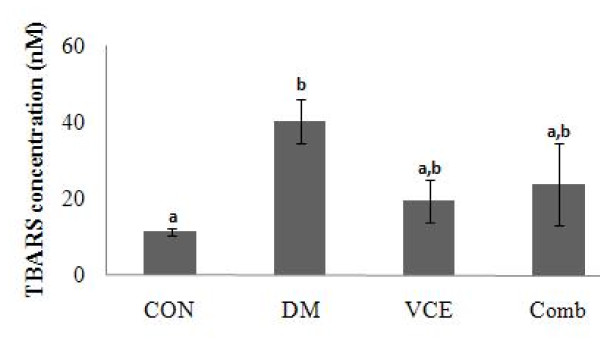
**Effect of dietary antioxidant supplementation on liver TBARS concentration (nM) in alloxan-induced diabetic mice**. Values are means ± SEM. Means at a time without a common letter differ, *P *< .05. (CON, control mice; DM, diabetic control mice; VCE, 0.5% vitamin C and 0.5% vitamin E supplemented diabetic mice; Comb, 0.5% vitamin C, 0.5% vitamin E, and 2.5% NAC supplemented diabetic mice).

Liver TBARS levels of the DM group were much higher than the CON group but they were not significantly reduced in the VCE and the Comb groups (Figure 2).

### Liver Vitamin E concentration

**Figure 3:**
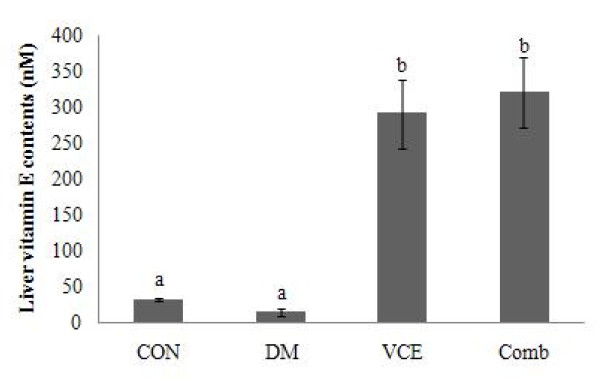
**Effect of dietary antioxidant supplementation on liver vitamin E contents (nM) in alloxan-induced diabetic mice**. Values are means ± SEM. Means at a time without a common letter differ, *P *< .05. (CON, control mice; DM, diabetic control mice; VCE, 0.5% vitamin C and 0.5% vitamin E supplemented diabetic mice; Comb, 0.5% vitamin C, 0.5% vitamin E, and 2.5% NAC supplemented diabetic mice).

Vitamin E levels of the DM group were not significantly different from those of the CON group. However, the levels of the vitamin E in the VCE and the Comb groups, supplemented with dietary antioxidants, including vitamin E, were significantly increased (13 folds) as compared to those of the DM group (Figure 3).

### Wound closure rate

**Figure 4:**
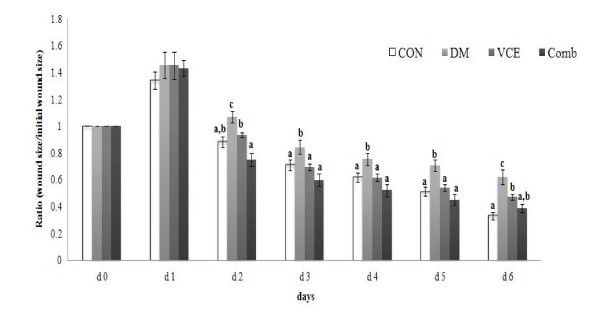
**Effect of dietary antioxidant supplementation on cutaneous wound closure rate in alloxan-induced diabetic mice**. The area of the wound of each time point was relative to the area of the wound on d 0 (set at 1.0). Values are means ± SEM. Means at a time without a common letter differ, *P *< 0.05. (CON, control mice; DM, diabetic control mice; VCE, 0.5% vitamin C and 0.5% vitamin E supplemented diabetic mice; Comb, 0.5% vitamin C, 0.5% vitamin E, and 2.5% NAC supplemented diabetic mice).

The wound closure rates of the DM group were significantly delayed from day 2 as compared to those of the CON group. However, the wound closure rates of the VCE and the Comb groups were accelerated significantly from day 2 as compared to those of the DM group (Figure 4).

### Relationships between wound size and blood glucose levels

The relative wound size (closure ratio) on days 6-8 after wounding (days 22-24 after alloxan injection) in each diet treatment group was positively correlated to the blood glucose levels on day 5 (starting day of antioxidant supplementation) up to day 24 (day 8 after wounding) after alloxan injection (Table 1).

**Table 1:** Relationships (Pearson's correlation coefficients) between wound size and blood glucose levels


		**Blood glucose levels**							
		**^§^d 5**	**d 9**	**d 12**	**d 15**	**d 16**	**d 18**	**d 21**	**d 24**

Wound size	^#^d 6	.434*	.468*	.595**	.531**	.553**	.562**	.620**	.575**
	d 7	.414*	.437*	.579**	.510*	.542**	.545**	.606**	.529*
	d 8	.340	.431*	.480*	.427*	.486*	.507*	.549**	.431

* *P *< .05, ** *P *< .01.

^§^day after alloxan injection, d 5: starting day of antioxidant supplementation, d 16: d 0 after wounding.

^#^day after wounding, d 8: d 24 after alloxan injection.

### The expression levels of oxidative stress and inflammatory response related proteins in mouse skin

**Figure 5:**
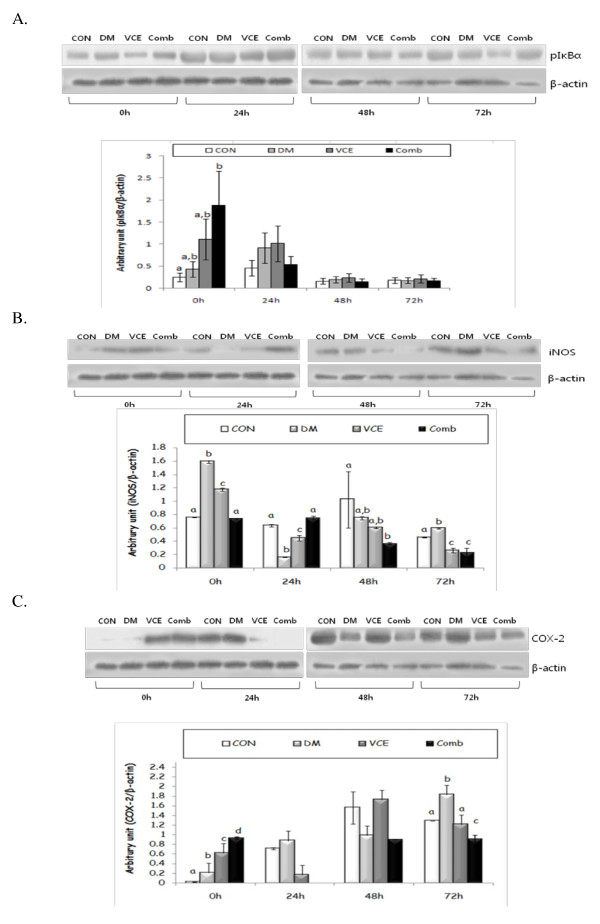
**Effect of dietary antioxidant supplementation on protein expression levels of pIκBα (A), inducible nitric oxide synthase (iNOS)(B), and cyclooxygenase (COX)-2(C) in cutaneous wounds of alloxan-induced diabetic mice**. Values are means ± SEM. Means for a variable without a common differ, *P *< .05. (CON, control mice; DM, diabetic control mice; VCE, 0.5% vitamin C and 0.5% vitamin E supplemented diabetic mice; Comb, 0.5% vitamin C, 0.5% vitamin E, and 2.5% NAC supplemented diabetic mice).

The protein levels of pIκBα showed a similar result to those of COX-2 at 0 h. However, there were no significant differences among all groups at 24 h, 48 h and 72 h (Figure 5A). The expression levels of inducible nitric oxide synthase (iNOS) in the DM group were significantly increased at basal level and steadily decreased after wounding. There was a significant decrease in iNOS protein levels in the VCE and the Comb groups as compared to those of the DM group at 72 h after wounding (Figure 5B). The expression levels of cyclooxygenase (COX)-2 protein were raised continuously in the DM group after wounding. The expression levels of COX-2 protein in the VCE group were comparable to those of the CON group at 72 h after wounding, and in the Comb group, the levels were maintained until 72 h after wounding (Figure 5C).

**Figure 6:**
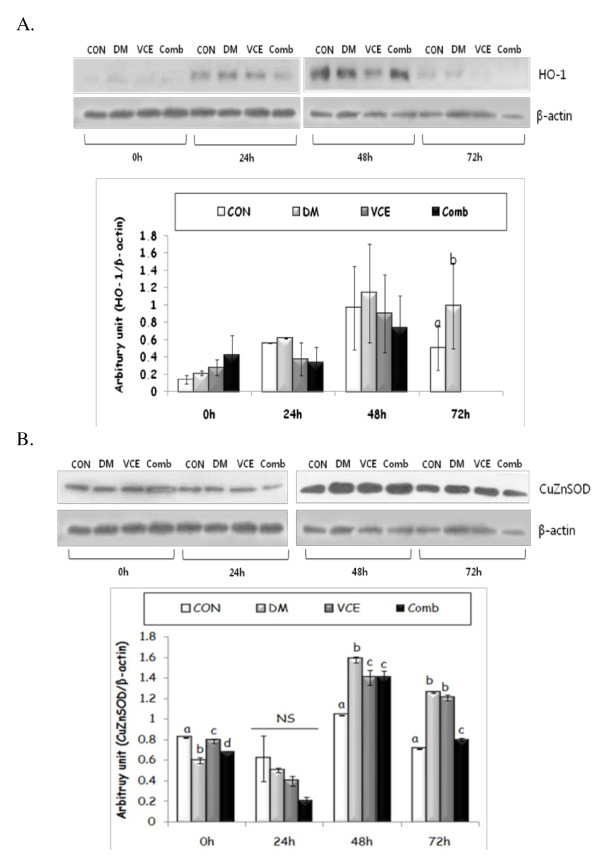
**Effect of dietary antioxidant supplementation on protein expression level of heme oxygenase (HO)-1(A) and copper zinc superoxide dismutase (CuZnSOD)(B) in cutaneous wounds of alloxan-induced diabetic mice**. Values are means ± SEM. Means for a variable without a common differ, *P *< .05. (CON, control mice; DM, diabetic control mice; VCE, 0.5% vitamin C and 0.5% vitamin E supplemented diabetic mice; Comb, 0.5% vitamin C, 0.5% vitamin E, and 2.5% NAC supplemented diabetic mice).

As shown in Figure 6A, the expression levels of heme oxygenase (HO)-1 protein at 72 h after wounding were remarkably increased in the DM group, as compared to those of the CON group, but the expression levels were barely detected in the dietary antioxidant supplemented groups, the VCE, and the Comb groups. The expression levels of CuZnSOD protein in the DM group at basal level were significantly lower, but at 48 h after wounding, the levels were higher than those of other antioxidant treatment groups (Figure 6B). In addition, the CuZnSOD protein levels of the Comb group were comparable to those of the CON group at 72 h after wounding.

## Discussion

The inflammatory stage is the most important in the wound healing process because the proper healing process requires recruitment of inflammatory cells and secretion of various mediators. However, an increase of oxidative stress, caused by wound occurrence with continuous hyperglycaemia in diabetes, prolongs inflammatory response and causes an ulcer by delaying a shift to the next steps. Therefore, controlling oxidative stress and inflammatory response through appropriate regulation of blood glucose levels is essential to accelerating the wound closure rate. We hypothesized that the regulation of blood glucose levels through dietary antioxidant supplementation reduces oxidative stress in the inflammatory stage and improves the early wound closure rate during cutaneous wound healing.

In previous studies, dietary antioxidant supplementation itself did not affect blood glucose levels [23], but this study showed a significant decreasing effect, as compared to the DM group, in spite of being at a higher than normal level. If dietary antioxidants are supplemented for a longer period, they might be effective in reducing blood glucose levels.

Oxidative stress is increased by enhancing the rate of ROS production and declining antioxidant defense in diabetes. Therefore, it makes it easier to cause oxidative damage in various tissues in diabetes. In this study, we confirmed that liver TBARS levels, the end product of lipid peroxidation, were increased in diabetes, but they were not significantly decreased by dietary antioxidant supplementation. This result stands in contrast with earlier works by Kim *et al*. [24] and Odetti *et al*. [25], showing decreasing TBARS levels in vitamin C or NAC supplemented diabetic mice. The contrasting result in this study may be associated with the short-term supplementation of dietary antioxidants. However, we found that kidney TBARS levels in diabetic mice were significantly decreased in the diabetic mice [26]. The results suggest a tissue-specific effect of dietary antioxidant supplementation on oxidative stress in diabetes.

The present study showed correlations between wound size and blood glucose levels after antioxidant supplementation. Blood glucose levels from the start day of diet treatment during the inflammatory stage affected relative wound size on days 6 to 8 after wounding. We suggested that it is possible to accelerate the wound closure rate by regulating blood glucose levels through dietary antioxidant supplementation, which attributed to lower oxidative stress, confirmed by decreased TBARS, HO-1 and CuZnSOD levels during the early inflammatory stage.

To examine the change of the inflammatory response by dietary antioxidant supplementation at molecular levels that modify the rate of the wound closure, we investigated the expressions of oxidative stress and inflammatory response related proteins. NFκB is composed of a family of inducible transcription factors that serve as essential regulators of the host immune and inflammatory response [27]. NFκB promotes the expression of enzymes that contribute to the pathogenesis of the inflammatory response, including the inducible form of nitric oxide synthase (iNOS) and inducible cyclooxygenase (COX-2) [28]. We evaluated the protein levels of pIκBα as an indirect method of determining NFκB expressions. The protein levels of pIκBα were comparable to those of COX-2, in that they showed significant differences among groups at 0 h, but there were no significant differences among groups at other time points. Heme oxygenase (HO)-1, an inducible form, is increased by various oxidative stress-inducing factors, such as NFκB. HO-1 has cytoprotective, proangiogenic, and anti-apoptotic properties as well as anti-inflammatory and antioxidant functions [29]. Our data showed raised levels of HO-1 in all experimental groups, including the CON group, at 48 h after wounding. However, the HO-1 levels in the dietary antioxidants supplemented groups, at 72 h after wounding, were hardly detected. The results suggest that dietary antioxidants may have some beneficial effects on shortening the inflammatory response via regulation of ROS production during the inflammatory stage, thus accelerating the entire wound healing rate.

Previous studies showed that SOD levels were decreased in diabetes by a deteriorated antioxidant defense system [30]. On the other hand, the SOD levels were increased in cutaneous wound healing [31]. The present result showed that, in the DM group, CuZnSOD expression levels were low at the baseline but increased at 48 h after wounding, which implies that oxidative stress is increased by continuous hyperglycaemia and wound occurrence in diabetes. Furthermore, the levels of CuZnSOD in the VCE and the Comb groups decreased at 48 h after wounding as compared to those of the DM group, showing a similar result in TBARS levels. This result suggests that ROS production was regulated by dietary antioxidant supplementation in the DM mice.

The levels of iNOS, one of the most important factors playing a key role in the wound healing process, increased in the early inflammatory stage after wounding and subsequently declined until the wound was healed. In this study, iNOS protein levels significantly increased at a basal level and decreased at 24 h after wounding, but again rose at 48 h and 72 h in the DM group. The results suggest that reduced iNOS protein levels at 24 h in the DM group were up-regulated due to increased oxidative stress caused by hyperglycaemia. Furthermore, increased iNOS protein levels, maintained until 72 h in the DM group, may decrease collagen synthesis, which plays an essential role in the proliferation and the remodeling stages next to the inflammatory stage, thus delaying the entire wound healing process. The expression levels of iNOS in the Comb group decreased gradually from 48 h, which paralleled the accelerated rate of wound closure on day 2. In particular, a significant decrease of the iNOS levels in the VCE and the Comb groups at 72 h, as compared to those of the DM group, influenced the improved wound closure rate, which showed a similar rate as the controls on day 3. Oxidative stress due to hyperglycaemia caused by diabetes increases secretion of various cytokines, which induce COX-2, a modulator of the inflammatory response in the biosynthesis of prostaglandins. Over-expression of the COX-2 protein decreases collagen synthesis and retards proliferation, thus delaying the entire cutaneous wound healing [32]. We found that COX-2 expression levels in the DM group gradually increased, which may prolong the inflammatory responses and delay wound healing. Significant decreased levels of the COX-2 at 72 h in the VCE group, as compared to those of the DM group, had a beneficial effect on the wound closure rate. Retained levels of COX-2 protein during the early inflammatory stage and significant lower levels at 72 h, as compared to those of other groups, may contribute to the advanced wound healing process related to the secretion of blood clotting factors [33] and shift to the proliferation and the remodeling stages.

Taken together, the results in this study suggest that dietary antioxidant supplementation creates synergetic effects that improve the condition of diabetes through the regulation of blood glucose levels and effects that accelerate the early inflammatory responses during cutaneous wound healing. Reduced iNOS and COX-2 expression levels may prevent prolonged inflammatory response by decreasing oxidative stress in dietary antioxidant supplementation, a fact that is confirmed by oxidative stress associated markers, namely, HO-1 and CuZnSOD.

## Conclusions

The current study demonstrated that the regulation of selective inflammatory mediators by dietary antioxidant cocktail supplementation selectively normalizes the early wound healing process in diabetes. Based on our results, antioxidant cocktail supplementation, including vit C, E and NAC, could be effectively used for diabetic wound healing therapy. However, our research was limited to animal studies; thus, it is important to determine the precise mechanisms in delayed wound healing for application to a human study. Furthermore, it is necessary to conduct a thorough investigation of each stage of the very intricate wound healing process that occurs in diabetes. Finally, further research should be conducted to verify a safe concentration of each antioxidant for clinical usage in diabetic patients.

## Competing interests

The authors declare that they have no competing interests.

## Authors' contributions

The author's responsibilities were as follows: NYP. performed the experiments and analysis and participated to data interpretation and manuscript writing. YL. contributed to study design, data interpretation and manuscript writing. All authors read and approved the final manuscript.
